# Peripheral blood transcriptomic clusters uncovered immune phenotypes of asthma

**DOI:** 10.1186/s12931-022-02156-w

**Published:** 2022-09-08

**Authors:** Hyun Woo Lee, Min-gyung Baek, Sungmi Choi, Yoon Hae Ahn, Ji-Young Bang, Kyoung-Hee Sohn, Min-Gyu Kang, Jae-Woo Jung, Jeong-Hee Choi, Sang-Heon Cho, Hana Yi, Hye-Ryun Kang

**Affiliations:** 1 Department of Internal Medicine, Seoul Metropolitan Government-Seoul National University Boramae Medical Center, Seoul, Korea; 2 Interdisciplinary Program in Precision Public Health, Korea University, Seoul, Korea; 3 Department of Internal Medicine, Seoul National University Hospital, 101 Daehak-ro, Jongno-Gu, Seoul, 110-744 Korea; 4 Department of Translational Medicine, Seoul National University College of Medicine, Seoul, Korea; 5 Department of Internal Medicine, Kyung Hee University Hospital, Seoul, Korea; 6 Department of Internal Medicine, Chungbuk National University College of Medicine, Chungbuk National University Hospital, Cheongju, Korea; 7 Department of Internal Medicine, Chung-Ang University College of Medicine, Seoul, Korea; 8 Department of Pulmonology and Allergy, Allergy and Clinical Immunology Research Center, Hallym University College of Medicine, Chuncheon, Korea; 9 Institute of Allergy and Clinical Immunology, Seoul National University Medical Research Center, Seoul National University College of Medicine, Seoul, Korea; 10 School of Biosystems and Biomedical Sciences, Korea University, 145 Anam-ro, Seongbuk-gu, Seoul, 02841 Korea

**Keywords:** Asthma, Cluster analysis, Microbiome, RNA-Seq, Transcriptome

## Abstract

**Background:**

Transcriptomic analysis has been used to elucidate the complex pathogenesis of heterogeneous disease and may also contribute to identify potential therapeutic targets by delineating the hub genes. This study aimed to investigate whether blood transcriptomic clustering can distinguish clinical and immune phenotypes of asthmatics, and microbiome in asthmatics.

**Methods:**

Transcriptomic expression of peripheral blood mononuclear cells (PBMCs) from 47 asthmatics and 21 non-asthmatics was measured using RNA sequencing. A hierarchical clustering algorithm was used to classify asthmatics. Differentially expressed genes, clinical phenotypes, immune phenotypes, and microbiome of each transcriptomic cluster were assessed.

**Results:**

In asthmatics, three distinct transcriptomic clusters with numerously different transcriptomic expressions were identified. The proportion of severe asthmatics was highest in cluster 3 as 73.3%, followed by cluster 2 (45.5%) and cluster 1 (28.6%). While cluster 1 represented clinically non-severe T2 asthma, cluster 3 tended to include severe non-T2 asthma. Cluster 2 had features of both T2 and non-T2 asthmatics characterized by the highest serum IgE level and neutrophil-dominant sputum cell population. Compared to non-asthmatics, cluster 1 showed higher *CCL23* and *IL1RL1* expression while the expression of *TREML4* was suppressed in cluster 3. *CTSD* and *ALDH2* showed a significant positive linear relationship across three clusters in the order of cluster 1 to 3. No significant differences in the diversities of lung and gut microbiomes were observed among transcriptomic clusters of asthmatics and non-asthmatics. However, our study has limitations in that small sample size data were analyzed with unmeasured confounding factors and causal relationships or function pathways were not verified.

**Conclusions:**

Genetic clustering based on the blood transcriptome may provide novel immunological insight, which can be biomarkers of asthma immune phenotypes.

*Trial registration *Retrospectively registered

**Supplementary Information:**

The online version contains supplementary material available at 10.1186/s12931-022-02156-w.

## Background

Therapeutic strategies for asthma have been established based on phenotypic features such as symptom severity and lung function [1]. However, conventional therapeutic approaches are often insufficient for adequate disease control, especially in severe cases due to the heterogeneous nature of asthma pathogenesis [2]. The asthma immune phenotypes have been used to explain the heterogeneity with various inflammatory and immunologic pathways involved in its pathogenesis [3]. For example, biomarkers of type 2 (T2) cell-mediated inflammation including eosinophils in sputum or blood cells, serum immunoglobulin E (IgE) levels, and fractional exhaled nitric oxide (FeNO) are used to identify potential candidates who may benefit from treatment with biologic agents targeting T2 inflammation. Recent clinical trials showed that anti-IL-4 or anti-IL-5 biologic agents successfully reduced the number of exacerbations and improved lung functions in T2 asthmatics [4, 5]. The conceptual classification of T2 and non-T2 asthma has led to a better understanding of the molecular mechanisms behind the heterogeneous phenotypes of asthma [6, 7]. Despite recent progress, the management of inhaled corticosteroid (ICS) refractory non-T2 asthma remains problematic, and the details of non-T2 asthma pathogenesis still need to be identified.

The transcriptome is a set of all the ribonucleic acid (RNA) transcripts for a specific biological condition. Transcriptome clustering has the potential to provide a deeper understanding of the biologic status [8]. Considering that over 80% of gene expressions are identified in peripheral blood mononuclear cells (PBMCs), transcriptomic analysis of peripheral blood samples is a relatively non-invasive, reproducible method of obtaining data. However, studies on transcriptomic cluster analyses using PBMCs are lacking in asthma patients. In addition, a recent study showed that transcriptome and microbiome profiles in the nasal epithelium of asthmatics were different from that of healthy controls, and these findings suggest that host-microbiome associations may exist in severe persistent asthma patients [9]. However, the association between the transcriptomic expressions of PBMCs and microbiome in the lung and gut has not been well evaluated in asthmatics.

In this study, we clustered asthmatics based on different blood transcriptomic expressions and investigated the clinical features, immune phenotypes, and microbiome compositions among clusters to gain a better understanding of asthma pathogenesis and to identify potential therapeutic targets.

## Methods

### Study design and eligibility criteria

In this cross-sectional study, we analyzed the medical records, transcriptome and microbiome data of the adult asthma cohort of asthmatics and non-asthmatic volunteers who visited thirteen medical centers across Korea from 2016 to 2019 [10]. All of the subjects in this cohort provided informed consent that was approved by the Institutional Review Board (IRB) of Seoul National University Hospital (IRB No. 1607-148-778). This study was conducted in compliance with the reporting of genetic association studies statement [11]. The transcriptome data extracted from PBMCs and microbiome data obtained from induced sputum and stool samples of all patients were included in the analysis.

Patients were diagnosed as asthmatics if they met the following criteria: (1) the presence of respiratory symptoms related to chronic airway inflammation including cough, sputum, dyspnea, and wheezing with (2) airway reversibility defined as a 12% and 200 mL or greater increase in forced expiratory volume in 1 s (FEV_1_) after the use of an inhaled short-acting bronchodilator or (3) airway hyperresponsiveness defined as a reduction of FEV_1_ 20% or more after inhalation of less than 16 mg/mL of methacholine [12].

Patients were considered to have severe asthma if they had reduced lung functions (FEV_1_ < 80% of predicted value) and asthma control questionnaire (ACQ) scores of 1.5 or higher with a daily inhaled corticosteroid (ICS) requirement equivalent to 1000 μg of beclomethasone or greater [13]. Patients who used antibiotics or experienced an acute exacerbation event within three months prior to the study were excluded.

### Assessment of clinical parameters

We obtained demographic and clinical information regarding age, sex, body mass index, smoking status, family history of asthma, pets, underlying conditions, results of lung function tests, and FeNO values. For asthmatics, we additionally obtained clinical information about asthma severity, levels of asthma control, ACQ-7 scores, asthma control test (ACT) scores, and doses of inhaled corticosteroids, oral steroids, long-acting beta2 agonists (LABA), long-acting muscarinic antagonists (LAMA), leukotriene receptor antagonists (LTRA), theophylline, omalizumab, and macrolides. To evaluate the immune phenotypes of asthmatics, sputum samples were counted for eosinophils (%), neutrophils (%), and macrophages (%). Blood samples were measured for total IgE, vitamin D, interferon (IFN)-γ, periostin, interleukin (IL)-4, IL-5, IL-13 and eosinophils (%).

### Samples and clinical parameters

We collected 2.5 mL of peripheral blood samples in the PAXgene Blood RNA Tube (BD Biosciences, San Jose, CA, USA) from each eligible patient. At least 2 mL of induced sputum samples were collected in the Falcon 50 mL conical centrifuge tube. We followed a standardized method to obtain induced sputum samples [14]. Sputum samples that contained squamous epithelial cells less than 20% of the total cell count were considered appropriate [15]. We collected at least 0.2 g of stool samples in a stool container (SPL Life Sciences, Pocheon-si, Korea). The collected blood, sputum, and stool samples were stored at − 70 °C.

### RNA extraction and transcriptome sequencing

RNA extraction was performed with a PAXgene Blood RNA kit IVD (QIAGEN, Hilden, Germany). The RNA-seq library was prepared with a TruSeq Stranded mRNA Sample Preparation Kit (Illumina, San Diego, CA, USA). The messenger RNA libraries were sequenced by paired-end 100 cycles on the HiSeq 2500 (Illumina). Low-quality bases in the raw reads were filtered out by FastQC ver 0.11.5 [16] and potentially existing sequencing adapters were trimmed with Skewer ver 0.2.2 [17]. The high-quality reads were mapped to the human reference genome hg19 (downloaded from UCSC genome browser, https://genome.ucsc.edu) by STAR ver 2.6 [18]. The gene expression level was quantified based on the aligned reads by Cufflinks package ver 2.2.1 [19]. The gene annotation of the reference genome was used as gene models and the expression values were calculated in Fragments Per Kilobase of transcript per Million fragments mapped (FPKM) unit.

### Clustering and transcriptome expression analysis

To identify any disease subsets within the asthmatic group, we explored the gene expression patterns using a hierarchical clustering method. The R-function “hclust” with the Euclidean distance and complete linkage option was used for the clustering analysis of gene expression profiles in FPKM values. The resultant clusters within the asthmatic group were designated as cluster 1, 2, and 3. The non-asthmatic group was not included in the clustering analysis and was regarded as an independent control cluster. The Principal Component Analysis (PCA) plots were generated using the R program. Heatmaps were plotted using the heatmap package and the Euclidean distance method. Different transcriptome expressions were analyzed, and comparisons were made among the three clusters as well as between asthmatics and non-asthmatics.

### Microbiome analysis

Metagenomic deoxyribonucleic acid (DNA) was extracted from 1 mL of induced sputum and 0.2 g of stool using a FastDNA SPIN Kit (MP Biomedicals, Irvine, CA, USA). The bacterial 16S ribosomal RNA (rRNA) gene was amplified using the extracted DNAs as templates. For the polymerase chain reaction, primers 341F and 805R and 2X KAPA HiFi HotStart ReadyMix (KAPA Biosystems, Roche, Wilmington, MA, USA) were used [20]. The sequencing library was constructed according to the 16S metagenomic sequencing library preparation methods (Illumina) [21]. The amplicon was purified using a XSEP MagBead (Celemics Inc., Seoul, Korea) and 300 bp paired-end sequencing using the MiSeq v3 platform (Illumina) was performed.

The resultant sequences were analyzed using Quantitative Insights in Microbial Ecology software (QIIME) [22] and the EzBiocloud 16S rRNA gene sequence database [10]. The beta-diversity was calculated using the weighted normalized UniFrac distance. The Principal coordinate analysis (PCoA) and PERMANOVA implemented in the Vegan package of the R software were used to compare the microbiome structures.

### Statistical analyses

The R statistical software, version 3.6.3 [R Core Team (2018), Vienna, Austria] was used for statistical analysis. A Log_2_ fold change > 2 or < -2 and a *q-value* < 0.05 were considered to be statistically significant. Categorical variables were analyzed with the Chi-square test and the Fisher’s exact test. Continuous variables were analyzed with the Kruskal–Wallis test. Differentially expressed genes (DEGs) were analyzed using the DESeq2 package [23]. The volcano plots for the expression-fold changes were drawn with the EnhancedVolcano package [24]. The difference of bacterial abundance was evaluated by Kruskal–Wallis test and Benjamini–Hochberg adjustment.

## Results

### Differences in asthmatics and non-asthmatics

A total of 46 asthmatic patients and 21 non-asthmatics met the eligibility criteria, and their baseline characteristics are summarized in Additional file 1: Table S1. Asthmatics were older, had more asthma-related comorbidities with worse lung functions, and had more frequent family histories of asthma.

### Transcriptomic clusters and their phenotypes in asthmatics

**Fig. 1:**
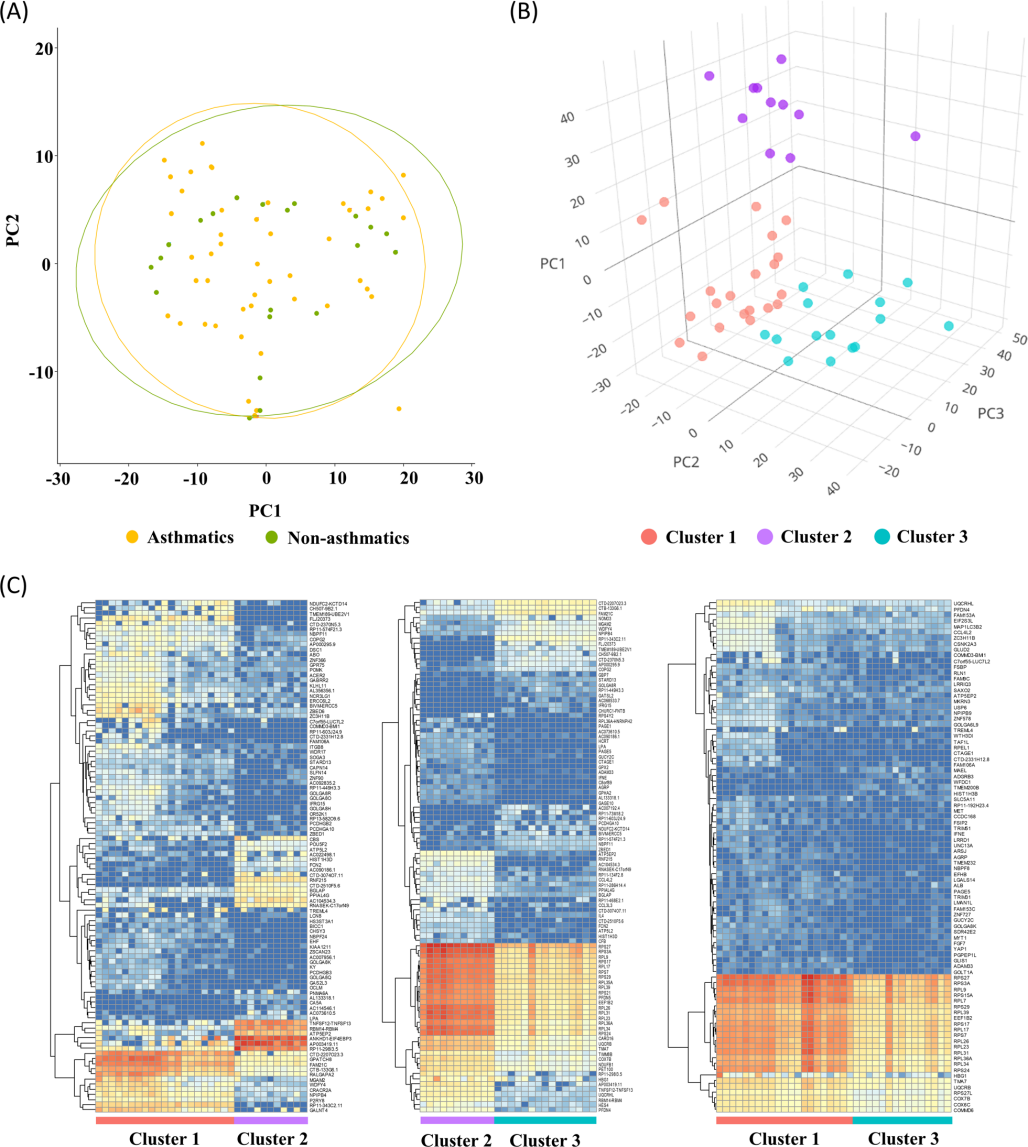
Distinctive patterns of transcriptomic expression of three clusters in asthmatics and non-asthmatics

A total of 19,841 transcriptomic expressions was observed in the blood samples. Although most transcriptomic expressions in PBMCs were similar in both asthmatics and non-asthmatics (Fig. 1A), *VSTM4* and *CCL23* were expressed significantly higher in asthmatics (*VSTM4*, log_2_ fold change = 5.14, q-value = 1.41 × 10^–5^; *CCL23*, log_2_ fold change = 2.41, q-value = 1.41 × 10^–5^) (Additional file 1: Table S2).

Among asthmatics, three distinct clusters were obtained based on the blood transcriptomic expressions: 21, 11, and 15 patients were grouped into three clusters, which were visualized through PCA (Fig. 1B). The heatmap for transcriptomic expression showed distinctive pattern expressions among the three clusters (Fig. 1C).

Phenotypic features of each cluster are described in Table 1. Phenotype analyses showed that the proportion of severe asthmatics was lowest at 28.6% in cluster 1 and highest in cluster 3 at 73.3%. Accordingly, cluster 3 involved more patients with an ACT score < 20 and had the lowest FEV_1_ and forced expiratory flow between 25 and 75% (FEF_25–75%_). The use of LAMA was found only in cluster 3. Cluster 1 had the highest levels of IL-5 and IL-13 while cluster 3 had the lowest levels of IL-5 and IL-13. There were no differences of serum IL-4 and IFN-γ levels among the three clusters. The blood IgE level and the sputum neutrophil count was highest in cluster 2.

**Table 1:** Baseline characteristics and clinical features of the asthmatics

	Cluster 1 (n = 21)	Cluster 2 (n = 11)	Cluster 3 (n = 15)	p-value
Age, mean (SD)	57.0 (8.5)	53.6 (6.9)	61.1 (7.4)	0.061
Female, n (%)	13 (61.9)	7 (63.6)	9 (60.0)	0.982
BMI, mean (SD)	24.1 (3.1)	24.0 (3.8)	24.5 (4.4)	0.947
Smoking, n (%)	5 (23.8)	0 (0.0)	5 (33.3)	0.113
PY ≥ 10, n (%)	1 (4.8)	0 (0.0)	4 (26.7)	0.047
Severe asthma (%)	6 (28.6)	5 (45.5)	11 (73.3)	0.029
Levels of asthma control				
Day symptom, n (%)	8 (38.1)	5 (45.5)	10 (66.7)	0.231
Limit of activity, n (%)	5 (23.8)	3 (27.3)	11 (73.3)	0.007
Night symptom, n (%)	5 (23.8)	4 (36.4)	7 (46.7)	0.355
SABA use for symptom control, n (%)	6 (28.6)	4 (36.4)	7 (46.7)	0.538
Acute exacerbation ≥ 2/year, n (%)	5 (23.8)	4 (36.4)	6 (40.0)	0.553
ACQ-7 score > 1.5, n (%)	6 (28.6)	3 (27.3)	8 (53.3)	0.245
ACT score < 20, n (%)	4 (19.0)	2 (18.2)	10 (66.7)	0.005
ICS dose				
High-dose, n (%)	1 (4.8)	2 (18.2)	4 (26.7)	0.180
Medium-dose, n (%)	5 (23.8)	6 (54.5)	8 (53.3)	0.113
Low-dose, n (%)	11 (52.4)	2 (18.2)	3 (20.0)	0.058
No ICS, n (%)	4 (19.0)	1 (9.1)	0 (0)	0.185
Oral steroid, n (%)	0 (0.0)	0 (0.0)	1 (6.7)	0.336
LABA, n (%)	16 (76.2)	10 (90.9)	15 (100.0)	0.099
LAMA, n (%)	0 (0.0)	0 (0.0)	4 (26.7)	0.009
LTRA, n (%)	13 (61.9)	8 (72.7)	7 (46.7)	0.392
Theophylline, n (%)	3 (14.3)	0 (0.0)	3 (20.0)	0.308
Omalizumab, n (%)	0 (0.0)	2 (18.2)	1 (6.7)	0.136
Macrolide, n (%)	0 (0.0)	0 (0.0)	2 (13.3)	0.108
Lung function				
FVC mL, mean (SD)	3,150 (730)	2,954 (940)	2,763 (581)	0.310
FVC %, mean (SD)	99 (16)	85 (15)	91 (16)	0.073
FEV_1_ mL, mean (SD)	2,382 (601)	2,110 (830)	1,936 (393)	0.100
FEV_1_%, mean (SD)	92 (18)	75 (23)	73 (11)	0.003
FEV_1_% < 80%, n (%)^a^	5 (23.8)	6 (54.5)	9 (60.0)	< 0.001
FEV_1_ /FVC %, mean (SD)	75 (6)	70 (13)	70 (9)	0.139
FEF_25-75_%, mean (SD)	68 (20)	54 (28)	43 (8)	0.001
FeNO ppb, mean (SD)	58 (54)	37 (23)	80 (20)	0.030
Blood				
IL-4, pg/mL, mean (SD)	111.5 (108.1)	72.5 (38.9)	83.7 (67.2)	0.405
IL-5, pg/mL, mean (SD)	10.9 (7.0)	7.8 (3.6)	4.8 (4.1)	0.008
IL-13, pg/mL, mean (SD)	5.0 (3.5)	3.6 (2.3)	2.2 (2.5)	0.027
Periostin, ng/mL, mean (SD)	53,644 (8943)	45,486 (13,949)	52,063 (13,706)	0.183
Interferon-γ, pg/mL, mean (SD)	19.7 (12.6)	17.5 (7.5)	17.0 (9.4)	0.714
White blood cell/uL, mean (SD)	6,607 (1804)	7,273 (1873)	7,239 (2827)	0.608
Eosinophil %, mean (SD)	4.6 (3.7)	2.8 (2.8)	5.9 (4.9)	0.152
Eosinophil/uL, mean (SD)	314 (293)	195 (182)	421 (339)	0.153
Total IgE, IU/mL, mean (SD)	361 (291)	768 (659)	311 (201)	0.009
Vitamin D, ng/mL, mean (SD)	20.2 (8.8)	19.5 (8.2)	19.7 (12.4)	0.980
Sputum				
Eosinophil %, mean (SD)	15.7 (17.5)	9.1 (3.8)	20.1 (12.4)	0.146
Neutrophil %, mean (SD)	0.7 (1.3)	16.0 (0.9)	6.0 (3.9)	< 0.001
Macrophage %, mean (SD)	63.9 (24.7)	74.3 (4.9)	56.1 (11.9)	0.051

^a^p-value was estimated using chi-squared test for trend in proportions

*ACQ* asthma control questionnaire, *ACT* asthma control test, *BMI* body mass index, *FeNO* fractional exhaled nitric oxide, *ICS* inhaled corticosteroid, *LAMA* acting muscarinic antagonist, *LTRA* leukotriene receptor antagonists, *FEF*_*25–75*_ forced expiratory flow between 25 and 75%, *FEV*_*1*_ forced expiratory volume in 1 s, *FVC* forced vital capacity, *PY* pack years, *SABA* short-acting beta-agonist, *SD* standard deviation

### Differential expression of transcriptome in asthmatic clusters compared to non-asthmatics

The 20 most differentially expressed transcriptomes between each asthmatic cluster and non-asthmatics were selected based on the absolute value of log_2_ fold changes, and these results are summarized in Table 2. Across the three clusters, the expression of *VSTM4* was consistently enhanced in each asthmatic cluster compared to non-asthmatics. In contrast, *PWP2* was commonly suppressed in all asthmatic clusters. Cluster-specifically, cluster 1 showed at least a twofold increase of *CCL23* and *IL1RL1* expression while the expression of *TREML4* was significantly suppressed in cluster 3.

**Table 2:** Top 20 transcriptomes differentially expressed between each cluster of asthmatics and non-asthmatics

Cluster 1 vs. Non-asthmatics			Cluster 2 vs. Non-asthmatics			Cluster 3 vs. Non-asthmatics		
Gene	Log2 fold change	q-value	Gene	Log2 fold change	q-value	Gene	Log2 fold change	q-value
*VSTM4*	4.97	4.83 × 10^–4^	*VSTM4*	4.62	1.21 × 10^–5^	*VSTM4*	5.58	1.48 × 10^–6^
*CCL23*	2.58	4.83 × 10^–4^	*WDFY4*	− 3.90	3.11 × 10^–11^	*RPL36A*	− 3.49	1.37 × 10^–7^
*IL1RL1*	2.05	9.16 × 10^–4^	*RP11-574F21.3*	− 3.90	5.80 × 10^–8^	*USP6*	− 3.50	1.37 × 10^–5^
*PWP2*	− 23.78	5.83 × 10^–12^	*GOLGA8O*	− 4.02	2.25 × 10^–4^	*CTAGE1*	− 3.51	8.35 × 10^–4^
			*TREML4*	− 4.32	0.010	*ATP5L2*	− 3.57	9.03 × 10^–4^
			*NCR3LG1*	− 4.39	3.39 × 10^–9^	*RNASEK-C17orf49*	− 3.61	0.010
			*IFRG15*	− 4.54	2.29 × 10^–4^	*PAGE1*	− 3.65	0.002
			*BIVM-ERCC5*	− 4.62	0.001	*RPL34*	− 3.67	4.16 × 10^–7^
			*C7orf55-LUC7L2*	− 4.62	0.009	*RPEL1*	− 3.71	0.004
			*RP11-603J24.9*	− 4.73	0.007	*RPL36A-HNRNPH2*	− 3.80	0.016
			*GOLGA8R*	− 4.83	2.22 × 10^–6^	*LPA*	− 4.06	0.003
			*AC007192.4*	− 4.89	0.036	*ZNF578*	− 4.22	1.09 × 10^–6^
			*CTD-2370N5.3*	− 5.35	4.12 × 10^–5^	*WTH3DI*	− 4.26	7.23 × 10^–5^
			*COMMD3-BMI1*	− 5.67	0.002	*UQCRHL*	− 4.39	8.03 × 10^–11^
			*RP11-449H3.3*	− 5.68	5.94 × 10^–5^	*ATP5EP2*	− 5.06	1.76 × 10^–7^
			*CH507-9B2.1*	− 6.97	1.92 × 10^–4^	*AC104534.3*	− 5.53	0.037
			*TMEM189-UBE2V1*	− 7.11	1.54 × 10^–8^	*KCNE1B*	− 5.84	0.003
			*PWP2*	− 7.32	0.045	*CTD-3074O7.11*	− 6.61	0.016
			*RP11-343C2.11*	− 8.14	8.46 × 10^–18^	*TREML4*	− 6.63	0.001
			*FLJ20373*	− 8.47	3.48 × 10^–10^	*PWP2*	− 23.00	8.03 × 10^–11^

If fold changes are upper than 1 (each cluster of asthmatics > non-asthmatics), then log2 fold change becomes positive

**Fig. 2:**
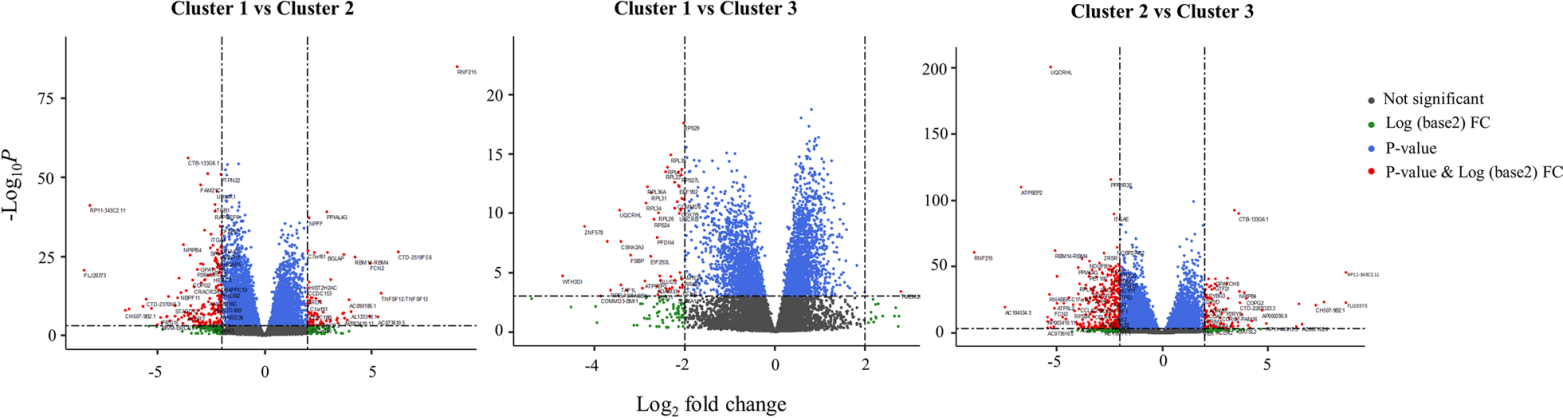
Volcano plots for differential expression of transcriptome among three clusters in asthmatics. *FC* fold change

The differential expression of transcriptomes among the three asthmatic clusters are summarized in Fig. 2. The 20 most differentially expressed transcriptomes based on the absolute value of log_2_ fold changes are described in Additional file 1: Table S3. Pre-specified genes not included in the top 20 genes of each cluster were compared among three clusters of asthmatics (Additional file 1: Table S4).

**Fig. 3:**
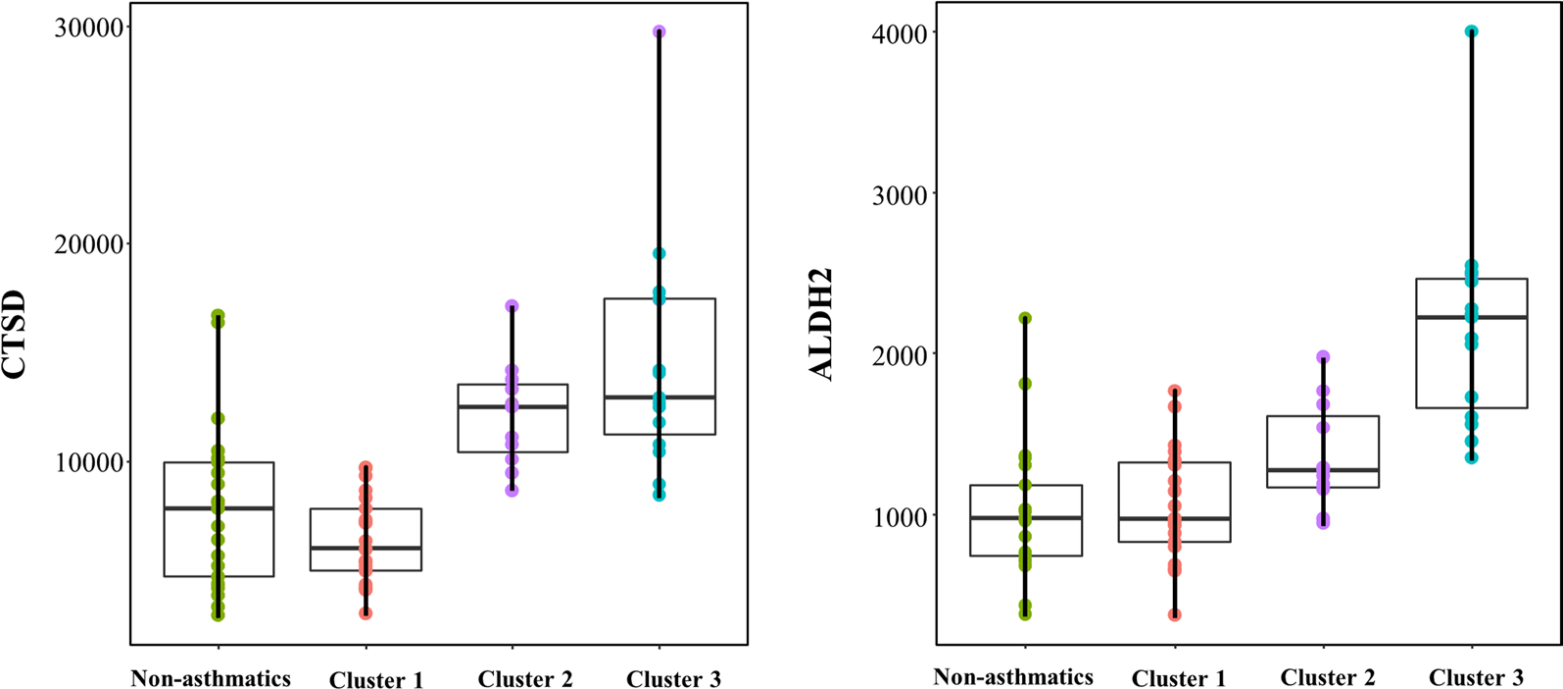
Linear regression analysis for transcriptome expression among three clusters in asthmatics and non-asthmatics

*CTSD* and *ALDH2* showed a significant positive linear relationship across three clusters in the order of cluster 1 to 3 in the linear regression model after Bonferroni correction (Fig. 3 and Additional file 1: Table S5).

### Lung and gut microbiomes in asthmatic clusters

**Fig. 4:**
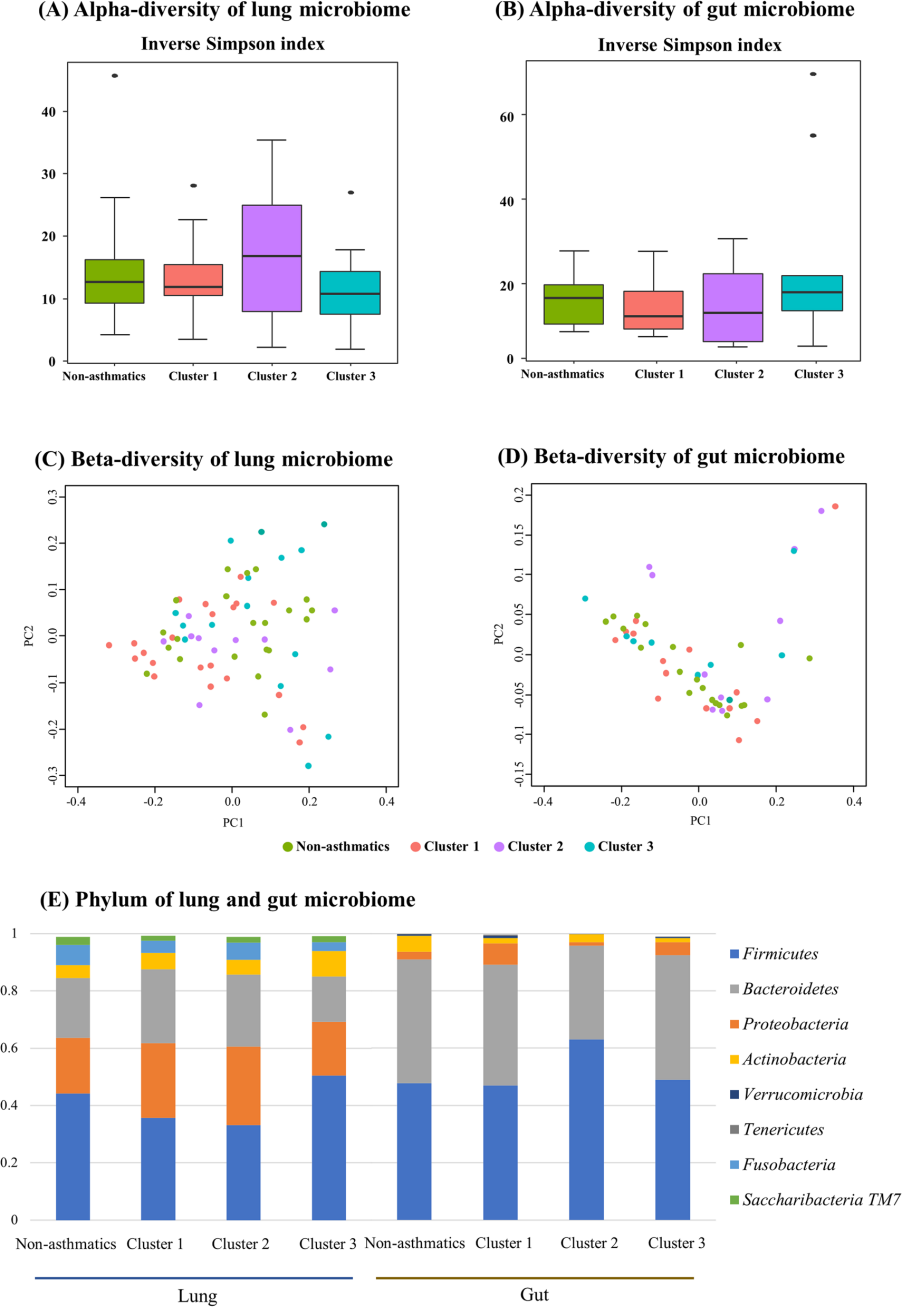
Alpha-/beta-diversity and phylum composition of lung and gut microbiomes in three clusters in asthmatics and non-asthmatics. **a** Alpha-diversity of lung microbiome, **b** Alpha-diversity of gut microbiome, **c** Beta-diversity of lung microbiome, **d** Beta-diversity of gut microbiome, **e** Phylum composition of lung and gut microbiome

Analyses of the alpha-diversity in the lung and the gut microbiomes did not show significant differences among three asthmatic clusters and non-asthmatics (Fig. 4). Analyses of the beta-diversity in the lung and gut microbiomes using the UniFrac distance showed no significant differences among three asthmatic clusters and non-asthmatics. Among lung clusters, *Streptococcus, Leptotrichia, Gemella, Alloprevotella, Granulicatella, Aggregatibacter* showed a difference in abundance compared to the non-asthmatics; however, these differences were attenuated after the Benjamini–Hochberg adjustment with the exception of *Alloprevotella* which revealed a significantly reduced abundance in cluster 3 (adjusted p-value = 0.049, Additional file 1: Table S6). The gut microbiome composition did not significantly differ among the three clusters and non-asthmatics.

## Discussion

Transcriptome analysis of PBMCs from asthmatics uncovered three distinct clusters through hierarchical clustering with different distributions in the PCA plot. The three clusters with different transcriptome expression profiles showed distinctive immunophenotypic features: cluster 1 was representative of non-severe T2 asthma, cluster 3 consisted of severe non-T2 asthmatics, and cluster 2 showed in-between clinical features with highest blood IgE and sputum neutrophil levels. These differences among clusters show that transcriptomic expressions of PBMCs may reflect the immunophenotypic features of asthmatics. However, we were unable to identify significant differences in the diversity of lung or gut microbiome among the three clusters in asthmatics. Therefore, transcriptomic expression of PBMCs may be largely unaffected by the diversity of lung and gut microbiomes.

As PBMCs are easily accessible through blood sampling and can reflect the presence of systemic inflammation, previous studies have evaluated the association between gene expression patterns in PBMCs and immune phenotypes in asthmatic patients. In one study, transcriptomic clustering analysis of PBMCs revealed systemic changes such as activation of innate immunity and antigen-independent T cell activation during acute exacerbation of asthma [25]. A clustering analysis based on transcriptomic profiles of PBMCs in asthmatic children identified a distinct cluster with a gene expression pattern associated with Th1 or Th17 inflammation and poor treatment outcomes [26]. In another study, severe asthmatics showed enhanced inflammatory mechanisms related to myeloid cell trafficking while lymphoid cell development in the same patients was attenuated [27]. Complementary to the findings of these previous studies, our study also showed that transcriptomic expression among the three clusters in adult asthmatics had distinct features related to T2 or non-T2 asthma. Therefore, transcriptome expression in PBMCs may predominantly show the immune phenotype of asthma while not including lung structural changes associated with asthma.

The expression of *VSTM4*, a B7-like protein that downregulates T cell activation [28], was higher in asthmatics compared to non-asthmatics regardless of cluster subtypes. Given that *VSTM4* reduces IFN-γ and IL-2 produced by T cells and inhibits naïve CD4^+^T cell differentiation into Th1 cells [28], *VSTM4* may contribute to a relative predominance of Th2 inflammation over Th1 inflammation in asthma. There may be a correlation between respiratory syncytial virus (RSV) infection and *VSTM4* in children. RSV showed a relationship with higher serum IgE levels and elevated Th2 inflammatory responses [29]. The causal relationship between expression of *VSTM4* and RSV infection is a topic that warrants further investigation.

*CCL23*, an eosinophilic-derived cytokine belonging to the CC chemokine family, has high chemotactic activity for resting T cells associated with eosinophilic airway inflammation [30, 31]. Although *CCL23* expression was increased in asthmatics compared to non-asthmatics, a higher transcriptomic expression of *CCL23* may be more closely related to T2 inflammatory features in cluster 1. As *CCL23* is produced and released from eosinophils, its expression is associated with increased eosinophilic inflammation [32, 33]. In nasal epithelial tissue and blood, higher expressions of *CCL23* were also reported in chronic eosinophilic rhinosinusitis [30, 34]. In our study, the expression of *CCL23* was higher in the PBMCs of cluster 1 compared to that of non-asthmatics. Similarly, the expression of *IL1RL1* was also higher in cluster 1 compared to non-asthmatics. *IL1RL1*, also known as ST2 which binds to IL-33, is associated with T2 inflammatory pathways and are upregulated in atopic asthma [35]. In bronchial epithelial cells of asthmatic patients, a higher expression of *IL1RL1* correlated with Th2 inflammation [36]. These results show that cluster 1 may be related to systemically enhanced Th2 inflammation caused by the activation of the IL-33/ST2 axis.

In our study, the severity of asthma increased with a linear trend of cathepsin D (CTSD) and aldehyde dehydrogenase 2 (*ALDH2)* expression from non-asthmatics to cluster 3. *CTSD*, a protease that breaks down abnormal or denatured proteins in airway, was more expressed with lung inflammation, especially in cystic fibrosis [37]. Considering that *CTSD* showed a potential role in the degradation of tumstatin that inhibits airway remodeling and airway hyperresponsiveness [38, 39], increased *CTSD* expression may be associated with severe asthma predominance in cluster 3. Deficiency in *ALDH2*, an enzyme that catalyzes the transformation from acetaldehyde to acetic acid, results in increased blood acetaldehyde levels and is related to the pathogenesis of alcohol-induced asthma [40]. In addition, *ALDH2* plays a protective role in the inflammatory damage caused by reactive oxygen species [41], which is known to be related with aggravated airway damage in asthma [42]. Therefore, a higher expression of *ALDH2* may be a protective response in asthmatics with high oxygen stress.

Gene expression patterns of airway structural cells are different to that of PBMCs. Among 90 DEGs in nasal epithelial cells and 4 DEGs in airway smooth muscle cells discovered in previous studies [43, 44], none were consistent with our findings using PBMCs. A recent study found 5 hub genes (*SERPINB2*, *SERPINB4*, *LTF*, *MUC5B*, and *CST4*) related to the pathogenesis of asthma in bronchial and nasal cells [45]. *SERPINB2* and *LTF* expressions in PBMCs were higher in cluster 3 compared to non-asthmatics and clusters 1 and 2. *SERPINB2* protects macrophages from apoptosis and regulates chemokines such as *CCL2* associated with monocyte and macrophage influx. As *SERPINB2* is upregulated during immune reactions to lipopolysaccharide, its high expression in non-T2 cluster 3 is not unexpected. Lactoferrin encoded by *LTF* is an immunomodulator with antimicrobial activity [46], and its increased expression may reflect the pathology of non-T2 neutrophilic asthma [47].

The relationship between transcriptomic profiles and the host microbiome is currently a topic of interest. Different *IL1A* expressions in airway epithelium of asthma patients were identified according to various microbiome profiles [48]. Transcriptomic profiles of PBMCs may provide a glimpse of systemic inflammatory features partly reflecting the inflammatory features in the lower respiratory tract in asthma [49]. We hypothesized that a relationship between the PBMC transcriptome and lung or gut microbiomes exists. However, we were unable to find a link between transcriptomic profiles in the peripheral blood and the diversity and taxonomy of microbiomes in the lung and gut. In fact, there have been studies that reported no significant differences in microbiome according to the phenotypes of asthma. The diversity and composition of lung and gut microbiome were broadly similar between asthmatics and non-asthmatics [50, 51]. In a comparison between patients with obstructive lung disease and control subjects, the diversity and composition of microbiome in BAL fluid were similar, when the samples were obtained using the same method [52]. The lack of such a relationship suggests that the immune response induced by the microbiome may be limited to the local environment.

This study has several limitations. First, the small sample size may have hindered the detection of important but small differences in immune phenotypes and microbiomes among clusters. Although the sputum eosinophil count, blood eosinophil count, and IL-4 levels seemed to differ among the three clusters, we could not find statistical significance behind these differences due to the large standard deviations. Second, due to the cross-sectional design of this study, we were unable to identify the causal relationship between transcriptomic profiles and clinical phenotypes or immune phenotypes. To prove the pathogenic causality, a longitudinal cohort study is needed. Third, confounding factors were not fully controlled. The results of our study should be interpreted with caution because different treatment regimens were used across the three clusters. Further studies with better control over confounding factors are needed. Fourth, although numerously different transcriptomic expressions were discovered among three clusters, we still do not know which inflammatory or functional pathways are responsible for the phenotypic differences among the clusters. Finally, caution is needed in generalizing our results, because validation analysis was not performed in a different cohort. Further external validation is necessary in a larger cohort of asthmatics.

## Conclusions

Transcriptomic profiles of PBMCs successfully differentiated asthmatics into three groups with different clinical and immune phenotypes, and differentially expressed genes may be potentially used as biomarkers representing asthma immune phenotypes.

## Supplementary Information

**Additional file 1: Table S1.** Baseline characteristics of the included asthmatics and non-asthmatics. BMI, body mass index; FeNO, fractional exhaled nitric oxide; FEF_25-75_, forced expiratory flow between 25–75%; FEV_1_, forced expiratory volume in 1 s; FVC, forced vital capacity; PY, pack years; SD, standard deviation. **Table S2. **Difference in transcriptome expression between asthmatic patients and non-asthmatic subjects. If fold changes are upper than 1 (each cluster of asthmatics > non-asthmatics), then log2 fold change becomes positive. **Table S3.** Top 20 transcriptomes differentially expressed among three clusters in asthmatics. If fold changes are upper than 1 (preceding cluster > following cluster), then log2 fold change becomes positive. **Table S4.** Pre-specified genes associated with asthma pathogenesis among three clusters in asthmatics. If fold changes are upper than 1 (preceding cluster > following cluster), then log2 fold change becomes positive. **Table S5.** Linear regression analysis for transcriptome expression among three clusters in asthmatics and non-asthmatics. ^a^ Bonferroni correction was conducted. **Table S6.** Lung and gut microbiome abundance in asthma patients classified by different three clusters. B-H, Benjamini-Hochberg.

## Data Availability

The datasets used or analyzed during the current study are available from the corresponding author on reasonable request.

## Abbreviations

ACQ: Asthma control questionnaire
ACT: Asthma control test
DEGs: Differentially expressed genes
DNA: Deoxyribonucleic acid
FEF_25–75_: Forced expiratory flow between 25 and 75%
FeNO: Fractional exhaled nitric oxide
FEV1: Forced expiratory volume in 1 s
FPKM: Fragments per kilobase of transcript per million fragments mapped
GWAS: Genome-wide association study
ICS: Inhaled corticosteroid
IRB: Institutional review board
LAMA: Acting muscarinic antagonist
LTRA: Leukotriene receptor antagonists
PBMCs: Peripheral blood mononuclear cells
PCA: Principal component analysis
PCoA: Principal coordinate analysis
RNA: Ribonucleic acid
T2: Type 2
