# Supplementary material for: Peripheral blood transcriptomic clusters uncovered immune phenotypes of asthma
Source: Respir Res. 2022 Sep 8;23:237. doi: 10.1186/s12931-022-02156-w (PMC9461267; doi:10.1186/s12931-022-02156-w)
Supplement: Supplementary file 1 — Additional file 1: Table S1. Baseline characteristics of the included asthmatics and non-asthmatics. BMI, body mass index; FeNO, fractional exhaled nitric oxide; FEF25-75, forced expiratory flow between 25–75%; FEV1, forced expiratory volume in 1 s; FVC, forced vital capacity; PY, pack years; SD, standard deviation. Table S2. Difference in transcriptome expression between asthmatic patients and non-asthmatic subjects. If fold changes are upper than 1 (each cluster of asthmatics > non-asthmatics), then log2 fold change becomes positive. Table S3. Top 20 transcriptomes differentially expressed among three clusters in asthmatics. If fold changes are upper than 1 (preceding cluster > following cluster), then log2 fold change becomes positive. Table S4. Pre-specified genes associated with asthma pathogenesis among three clusters in asthmatics. If fold changes are upper than 1 (preceding cluster > following cluster), then log2 fold change becomes positive. Table S5. Linear regression analysis for transcriptome expression among three clusters in asthmatics and non-asthmatics. a Bonferroni correction was conducted. Table S6. Lung and gut microbiome abundance in asthma patients classified by different three clusters. B-H, Benjamini-Hochberg. [file 12931_2022_2156_MOESM1_ESM.docx]

**Additional file 1**

**Table S1. Baseline characteristics of the included asthmatics and non-asthmatics**

|  | **Asthmatics (n=47)** | **Non-asthmatics (n=21)** | **p-value** |
| --- | --- | --- | --- |
| **Age, mean (SD)** | 57.5 (8.1) | 49.3 (8.8) | < 0.001 |
| **Female, n (%)** | 29 (61.7) | 14 (66.7) | 0.904 |
| **BMI, mean (SD)** | 24.2 (3.7) | 23.9 (3.0) | 0.733 |
| **Smoking, n (%)** | 10 (21.3) | 2 (9.5) | 0.406 |
| **PY ≥10, n (%)** | 1 (4.8) | 5 (10.6) | 0.774 |
| **Family history of asthma, n (%)** | 15 (31.9) | 0 (0.0) | 0.009 |
| **Pet, n (%)** | 5 (10.6) | 1 (4.8) | 0.744 |
| **Underlying condition** |  |  |  |
| Allergic rhinitis, n (%) | 34 (72.3) | 0 (0.0) | < 0.001 |
| Chronic rhinosinusitis, n (%) | 23 (48.9) | 0 (0.0) | < 0.001 |
| Allergic conjunctivitis, n (%) | 2 (4.3) | 0 (0.0) | 0.855 |
| Nasal polyp, n (%) | 8 (17.0) | 0 (0.0) | 0.108 |
| Angioedema, n (%) | 1 (2.1) | 0 (0.0) | 1.000 |
| Chronic urticaria, n (%) | 3 (6.4) | 0 (0.0) | 0.586 |
| Drug allergy, n (%) | 2 (4.3) | 0 (0.0) | 0.855 |
| Food allergy, n (%) | 1 (2.1) | 0 (0.0) | 1.000 |
| Diabetes mellitus, n (%) | 6 (12.8) | 0 (0.0) | 0.211 |
| Hypertension, n (%) | 6 (12.8) | 2 (9.5) | 1.000 |
| **Lung function** |  |  |  |
| FVC, mL, mean (SD) | 2,981 (745) | 3,224 (620) | 0.196 |
| FVC, %, mean (SD) | 93 (17) | 103 (10) | 0.013 |
| FEV_1_, mL, mean (SD) | 2,176 (627) | 2,677 (552) | 0.002 |
| FEV_1_, %, mean (SD) | 82 (20) | 100 (9) | < 0.001 |
| FEV_1_/FVC, %, mean (SD) | 72 (9) | 81 (11) | 0.001 |
| FEF_25-75_, %, mean (SD) | 57 (22) | 76 (14) | < 0.001 |
| FeNO, ppb, mean (SD) | 60 (42) | 18 (2) | < 0.001 |

SD: standard deviation

**Table S2. Difference in transcriptome expression between asthmatic patients and non-asthmatic subjects**

| **Gene name** | **Gene symbol** | **Biologic process** | **Function** | **Asthmatics *vs.* non-asthmatics** | | |
| --- | --- | --- | --- | --- | --- | --- |
|  |  |  |  | **Log2 fold change** | **p-value** | **q-value** |
| V-Set and Transmembrane Domain Containing 4 | VSTM4 | Calcium channel modulation | Negative regulator of T cells | 5.14 | 1.97x10^-9^ | 1.41x10^-5^ |
| C-C Motif Chemokine Ligand 23 | CCL23 | Monocyte chemotaxis / Cellular calcium ion homeostasis | Chemotactic activity for monocytes, resting T-lymphocytes, and neutrophils | 2.41 | 1.48x10^-9^ | 1.41x10^-5^ |

If fold changes are upper than 1 (each cluster of asthmatics> non-asthmatics), then log2 fold change becomes positive.

**Table S3. Top 20 transcriptomes differentially expressed among 3 clusters in asthmatics**

| **Cluster 1 vs. Cluster 2** | | | **Cluster 2 vs. Cluster 3** | | | **Cluster 1 vs. Cluster 3** | | |
| --- | --- | --- | --- | --- | --- | --- | --- | --- |
| **Gene** | **Log2 fold change** | **Q-value** | **Gene** | **Log2 fold change** | **Q-value** | **Gene** | **Log2 fold change** | **Q-value** |
| *RNF215* | 9.03 | 1.41x10^-81^ | *FLJ20373* | 8.72 | 3.90x10^-22^ | *RP11-192H23.4* | -2.84 | 0.005 |
| *CTD-2510F5.6* | 6.27 | 4.24x10^-25^ | *RP11-343C2.11* | 8.7 | 6.71x10^-44^ | *RPL34* | -2.85 | 1.18x10^-9^ |
| *TNFSF12-TNFSF13* | 5.45 | 9.27x10^-13^ | *TMEM189-UBE2V1* | 7.68 | 2.73x10^-22^ | *FAM153C* | -2.86 | 0.006 |
| *AC073610.5* | 5.32 | 8.73x10^-6^ | *CH507-9B2.1* | 7.3 | 2.15x10^-20^ | *ATP5EP2* | -2.89 | 3.58x10^-4^ |
| *FCN2* | 4.95 | 8.15x10^-22^ | *AC007192.4* | 6.64 | 8.10x10^-7^ | *CCDC168* | -2.93 | 0.001 |
| *AC104534.3* | 4.9 | 4.23x10^-6^ | *CTD-2370N5.3* | 6.48 | 4.88x10^-21^ | *TRIM51* | -2.94 | 0.015 |
| *GOLGA8O* | -4.58 | 7.95x10^-6^ | *RP11-73M18.2* | 6.36 | 9.02x10^-5^ | *LRRD1* | -2.97 | 0.015 |
| *CTD-2331H12.8* | -4.77 | 0.027 | *RBM14-RBM4* | -5.06 | 1.04x10^-59^ | *FSBP* | -3.2 | 5.25x10^-6^ |
| *BIVM-ERCC5* | -4.8 | 3.15 x10^-4^ | *FCN2* | -5.09 | 9.79x10^-18^ | *CTAGE1* | -3.28 | 0.002 |
| *IFRG15* | -4.86 | 9.30x10^-6^ | *RPL36A-HNRNPH2* | -5.14 | 1.15x10^-4^ | *CSNK2A3* | -3.41 | 5.22x10^-7^ |
| *COMMD3-BMI1* | -5.01 | 0.002 | *LPA* | -5.25 | 8.12x10^-10^ | *TAF1L* | -3.41 | 6.63x10^-4^ |
| *RP11-603J24.9* | -5.04 | 0.005 | *UQCRHL* | -5.28 | 7.67x10^-197^ | *UQCRHL* | -3.43 | 3.45x10^-9^ |
| *GOLGA8R* | -5.29 | 2.14 x10^-8^ | *CTD-3074O7.11* | -5.28 | 9.60x10^-4^ | *RPEL1* | -3.64 | 0.002 |
| *C7orf55-LUC7L2* | -5.41 | 0.003 | *RNASEK-C17orf49* | -5.33 | 4.68x10^-28^ | *ZC3H11B* | -3.71 | 5.05x10^-7^ |
| *CTD-2370N5.3* | -5.53 | 3.77x10^-11^ | *CTD-2510F5.6* | -5.42 | 1.59x10^-8^ | *COMMD3-BMI1* | -3.85 | 0.004 |
| *RP11-449H3.3* | -5.7 | 5.50x10^-9^ | *AC073610.5* | -5.42 | 7.62x10^-4^ | *C7orf55-LUC7L2* | -3.97 | 0.024 |
| *TMEM189-UBE2V1* | -6.32 | 4.10x10^-8^ | *AP003419.11* | -5.44 | 1.94x10^-11^ | *ZNF578* | -4.21 | 4.80x10^-8^ |
| *CH507-9B2.1* | -6.53 | 9.41x10^-8^ | *ATP5EP2* | -6.67 | 6.94x10^-107^ | *CTD-2331H12.8* | -4.51 | 0.028 |
| *RP11-343C2.11* | -8.18 | 5.42x10^-39^ | *AC104534.3* | -7.46 | 9.12x10^-19^ | *WTH3DI* | -4.7 | 1.55x10^-84^ |
| *FLJ20373* | -8.44 | 1.17x10^-19^ | *RNF215* | -8.89 | 2.79x10^-58^ | *TREML4* | -5.39 | 0.007 |

If fold changes are upper than 1 (preceding cluster > following cluster), then log2 fold change becomes positive.

**Table S4. Pre-specified genes associated with asthma pathogenesis among 3 clusters in asthmatics**

| **Gene name** | **Gene symbol** | **Biological process** | **Function** | **Comparison** | **Log2 fold change** | **q-value** |
| --- | --- | --- | --- | --- | --- | --- |
| Gasdermin B | *GSDMB* | Programmed cell death / defense response to bacterium / cytolysis | Host defense against pathogen infection and danger signals | Cluster 3 vs. Cluster 2 | 1.00 | 1.61x10^-7^ |
| Glucocorticoid Induced 1 | *GLCCI1* | Unknown | Modulating glucocorticoid sensitivity | Cluster 2 vs. Cluster 1 | 1.29 | 3.48x10^-9^ |
|  |  |  |  | Cluster 2 vs. Cluster 3 | 1.11 | 1.62x10^-7^ |
| IL-1 Receptor-Like 1 | *IL1RL1* | Negative regulation of T-helper 1 type immune response | ST2, IL-33 receptor | Cluster 2 vs. Cluster 1 | 1.61 | 0.002 |
|  |  |  |  | Cluster 2 vs. Cluster 3 | 0.90 | 0.017 |
| IL-5 Receptor, alpha | *IL5RA* | Inflammatory response to antigenic stimulus / Cytokine-mediated signaling pathway | IL-5 receptor activity | Cluster 2 vs. Cluster 1 | 0.91 | 0.010 |
|  |  |  |  | Cluster 2 vs. Cluster 3 | 0.78 | 0.034 |
| IL-6 Receptor | *IL6R* | Neutrophil mediated immunity / Monocyte chemotaxis | IL-6 receptor activity / IL-11 receptor activity | Cluster 2 vs. Cluster 1 | 0.41 | 0.049 |
|  |  |  |  | Cluster 2 vs. Cluster 3 | 1.16 | 1.84x10^-7^ |

If fold changes are upper than 1 (preceding cluster > following cluster), then log2 fold change becomes positive.

IL: interleukin

**Table S5. Linear regression analysis for transcriptome expression among 3 clusters in asthmatics and non-asthmatics**

| **Gene name** | **Gene symbol** | **Biological process** | **Function** | **Beta-coefficient** | **p-value** | **Adjusted p-value^a^** |
| --- | --- | --- | --- | --- | --- | --- |
| Cathepsin D | CTSD | Metabolic degradation of intracellular proteins / regulation of programmed cell death | Extracellular matrix remodeling | 2,200 | <0.001 | 0.037 |
| Aldehyde Dehydrogenase 2 | ALDH2 | Catalyze the transformation from acetaldehyde to acetic acid | Protection against oxidative stress | 334 | <0.001 | 0.016 |

^a^ Bonferroni correction was conducted.

**Table S6. Lung and gut microbiome abundance in asthma patients classified by different 3 clusters**

| **Lung microbiome** | **Kruskal–Wallis one-way analysis of variance** | | **Gut microbiome** | **Kruskal–Wallis one-way analysis of variance** | |
| --- | --- | --- | --- | --- | --- |
|  | **p-value** | **B-H p-value** |  | **p-value** | **B-H p-value** |
| **Phylum** |  |  | **Phylum** |  |  |
| Firmicutes | 0.035* | 0.453 | Firmicutes | 0.083 | 0.311 |
| Proteobacteria | 0.217 | 0.478 | Bacteroidetes | 0.394 | 0.492 |
| Bacteroidetes | 0.130 | 0.478 | Proteobacteria | 0.193 | 0.401 |
| Actinobacteria | 0.922 | 0.922 | Actinobacteria | 0.078 | 0.311 |
| Fusobacteria | 0.174 | 0.478 | Verrucomicrobia | 0.065 | 0.311 |
| Saccharibacteria (TM7) | 0.492 | 0.640 | Tenericutes | 0.362 | 0.492 |
| Spirochaetes | 0.173 | 0.478 | Euryarchaeota | 0.113 | 0.340 |
| SR1 | 0.690 | 0.801 | Fusobacteria | 0.235 | 0.401 |
| Tenericutes | 0.429 | 0.619 | Streptophyta | 0.353 | 0.492 |
| Synergistetes | 0.257 | 0.478 | Cyanobacteria | 0.547 | 0.586 |
| Peregrinibacteria | 0.235 | 0.478 | Lentisphaerae | 0.046* | 0.311 |
| Chloroflexi | 0.361 | 0.586 | Saccharibacteria (TM7) | 0.754 | 0.754 |
| Cyanobacteria | 0.739 | 0.801 | Synergistetes | 0.143 | 0.356 |
|  |  |  | Spirochaetes | 0.501 | 0.578 |
|  |  |  | Planctomycetes | 0.241 | 0.401 |
| **Genus** |  |  | **Genus** |  |  |
| *Streptococcus* | 0.049* | 0.444 | *Bacteroides* | 0.398 | 0.728 |
| *Prevotella* | 0.167 | 0.664 | *Prevotella* | 0.566 | 0.734 |
| *Neisseria* | 0.102 | 0.591 | *Faecalibacterium* | 0.961 | 0.968 |
| *Veillonella* | 0.129 | 0.637 | *Ruminococcus* | 0.235 | 0.720 |
| *Haemophilus* | 0.785 | 0.815 | *Clostridium* | 0.062 | 0.720 |
| *Porphyromonas* | 0.458 | 0.664 | *Romboutsia* | 0.107 | 0.720 |
| *Fusobacterium* | 0.187 | 0.664 | *Alistipes* | 0.038* | 0.720 |
| *Rothia* | 0.534 | 0.673 | *Eubacterium* | 0.126 | 0.720 |
| *Saccharimonas* | 0.479 | 0.664 | *Blautia* | 0.704 | 0.797 |
| *Actinomyces* | 0.779 | 0.813 | *Phascolarctobacterium* | 0.095 | 0.720 |
| *Leptotrichia* | 0.009* | 0.145 | *Escherichia* | 0.295 | 0.728 |
| *Gemella* | 0.029* | 0.348 | *Barnesiella* | 0.041* | 0.720 |
| *Pseudomonas* | 0.055 | 0.449 | *Bifidobacterium* | 0.062 | 0.720 |
| *Campylobacter* | 0.019* | 0.252 | *Lactobacillus* | 0.152 | 0.720 |
| *Alloprevotella* | 0.001* | 0.049* | *Anaerostipes* | 0.729 | 0.808 |
| *Capnocytophaga* | 0.107 | 0.605 | *Roseburia* | 0.233 | 0.720 |
| *Lautropia* | 0.481 | 0.664 | *Megamonas* | 0.088 | 0.720 |
| *Bacteroides* | 0.124 | 0.637 | *Subdoligranulum* | 0.790 | 0.832 |
| *Tropheryma* | 0.666 | 0.747 | *Streptococcus* | 0.043* | 0.720 |
| *Actinobacillus* | 0.741 | 0.780 | *Lachnospira* | 0.856 | 0.884 |
| *Parvimonas* | 0.931 | 0.939 | *Oscillibacter* | 0.249 | 0.722 |
| *Treponema* | 0.165 | 0.664 | *Parabacteroides* | 0.365 | 0.728 |
| *Granulicatella* | 0.005* | 0.092 | *Collinsella* | 0.011* | 0.720 |
| *Megasphaera* | 0.141 | 0.664 | *Dialister* | 0.459 | 0.734 |
| *Oribacterium* | 0.219 | 0.664 | *Agathobacter* | 0.675 | 0.773 |
| *Peptostreptococcus* | 0.059 | 0.471 | *JN713389* | 0.121 | 0.720 |
| *Atopobium* | 0.156 | 0.664 | *Alloprevotella* | 0.065 | 0.720 |
| *PAC000661* | 0.542 | 0.675 | *Fusicatenibacter* | 0.258 | 0.728 |
| *Selenomonas* | 0.171 | 0.664 | *CCMM* | 0.312 | 0.728 |
| *Aggregatibacter* | 0.015* | 0.205 | *Coprococcus* | 0.034* | 0.720 |

B-H, Benjamini-Hochberg

*<0.05
